# Efficiency of Orthodontic Adhesives: Influence of Saliva and Shear Direction—In Vitro Study

**DOI:** 10.3390/jfb17020089

**Published:** 2026-02-11

**Authors:** Tatiana Ignatova-Mishutina, Elena Xuriguera, Nuno Gustavo d’Oliveira, Meritxell Sánchez-Molins

**Affiliations:** 1 Faculty of Dentistry, Universidad de Barcelona, Carrer de la Feixa Llarga, s/n, L’Hospitalet de Llobregat, 08907 Barcelona, Spain; tim.ortodoncia@gmail.com (T.I.-M.); meritxellsanchez@ub.edu (M.S.-M.); 2 Faculty of Chemistry, Universidad de Barcelona, Carrer Martí i Franquès 1, 08028 Barcelona, Spain; xuriguera@ub.edu

**Keywords:** shear bond strength, adhesive remnant index, hydrophilic adhesive, self-etch adhesive, bracket bonding

## Abstract

This in vitro study evaluated the shear bond strength (SBS) and adhesive remnant index (ARI) of orthodontic molar tubes bonded using conventional, hydrophilic, and self-etch adhesives under dry and saliva-contaminated conditions, while also assessing the impact of shear force direction. Extracted molars were bonded with Transbond XT™ (T), Transbond MIP™ (M), or Scotchbond Universal™ (S) under dry or saliva-contaminated conditions. Debonding was performed at 90° or 45°, introducing a clinically relevant but underexplored variable in orthodontic bond-strength testing. ARI scores were assessed via stereomicroscopy and visual inspection. Statistical tests (Kruskal–Wallis and Mann–Whitney) showed no significant SBS differences among adhesives under identical conditions (*p* > 0.05). However, all adhesives exhibited significantly reduced SBS under saliva contamination (*p* < 0.001; T: 5.4 vs. 4.1 MPa; M: 5.7 vs. 3.6 MPa; S: 5.5 vs. 4.5 MPa). In dry conditions, SBS was significantly higher with 45° debonding (*p* < 0.05). Under contamination, SBS varied by ARI score (*p* = 0.05), with ARI 0 specimens showing higher SBS than ARI 3. These findings confirm that moisture reduces bond strength across adhesive types, while 45° force application enhances SBS under dry conditions. ARI score variability under contamination may reflect complex failure modes.

## 1. Introduction

Success in fixed orthodontics relies on effective dental movements, minimal dental chair time, and the absence of treatment delays due to unwanted bracket or tube debonding. Bracket bonding, pioneered by Newman [1], transfers the forces generated by archwires and auxiliary appliances to the teeth through the bracket base. Consequently, efficient adhesion is critical for achieving successful treatment outcomes, as unwanted debonding can lead to delays, increased costs, and patient inconvenience [2]. Factors that influence adhesion encompass enamel adhesive type, acid concentration, etching time, adhesive composition, bracket material and base design, tooth surface characteristics, oral environment, and skill of the clinician [3,4,5].

Shear bond strength (SBS) is a fundamental in vitro parameter used to characterize orthodontic bonding materials. It represents the maximum force required to debond a bracket, expressed in Newtons and normalized to the bracket base surface area. Clinically acceptable SBS values, sufficient to resist the oral environment, masticatory and orthodontic forces while allowing safe bracket removal without enamel damage, typically range from 5.9 to 7.8 MPa [6,7]. Although numerous studies have assessed SBS, most evaluate only a single 90° load applied to the occlusal aspect of the bracket. It is important to acknowledge, however, that forces generated during mastication and orthodontic treatment are multidirectional—an aspect largely neglected in previous research.

SBS is intrinsically related to bracket design, encompassing both the characteristics of the base surface and the materials used in its manufacture [5]. Previous studies have shown that ceramic brackets generally exhibit higher SBS values than metallic brackets; however, this apparent advantage may be offset by the increased risk of enamel damage during debonding, as greater force is required to remove them. In the case of molars, orthodontists often prefer bonding molar tubes rather than using molar bands, owing to their cost-effectiveness, smaller inventory requirements, improved accuracy in tube positioning, and reduced chair time [8,9,10]. Tubes additionally present several advantages over traditional molar bands, such as reduced clinical placement time, improved preservation of periodontal health through easier hygiene and maintenance of biological distances, and the elimination of the need for interdental separation [11]. Nevertheless, molar tubes have been reported to present a higher failure rate [10]. Positioned posteriorly, these tubes are subjected to stronger masticatory forces and are more prone to bonding challenges, such as saliva contamination, which is more difficult to control in this region. Thus, although some studies have reported higher in vitro SBS values for molar tubes [11], clinical complications during bonding or the adverse intraoral environment may increase their likelihood of debonding—an aspect investigated in the present study.

Achieving adequate SBS and minimizing unintended bracket detachment during bonding or treatment critically depends on the choice of adhesive. An ideal adhesive system should reliably bond orthodontic appliances to the tooth surface, provide sufficient working time for accurate placement, be biocompatible, tolerate moisture or other contaminants, maintain adequate retention against normal oral forces, and effectively transmit the intended orthodontic forces to the teeth [12]. Additionally, post-treatment removal of the adhesive should be straightforward, without causing damage to the enamel or underlying tooth structure [6].

The application of acid etching, first introduced by Buonocore, is a well-established method for enamel conditioning in orthodontic bracket bonding [13,14]. Etching dissolves the outer enamel layer, transforming the surface from a hydrophobic, low-energy state to a hydrophilic, high-energy state, thereby increasing surface tension and wettability. This enhances resin penetration into the enamel, providing mechanical retention and higher SBS values [6]. Shorter etching times, such as 15 s, may appear more conservative than the honeycomb pattern observed with 30 s etching [15,16,17]; alternative approaches include self-etch adhesives, which produce a milder etch pattern and shallower enamel penetration than 37% phosphoric acid [18,19]. In the self-etch technique, conditioning and priming are combined in a single acidic solution, eliminating the etching and rinsing steps, which reduces procedural errors such as moisture contamination [20,21], decreases aerosol generation, saves time, and minimizes enamel loss while maintaining clinically acceptable SBS [14,22]. However, SBS of self-etching adhesives is controversial. Some studies report that conventional etching produces longer and more extensive resin tags (11.09 ± 2.80 μm) than self-etch adhesives (6.30 ± 1.85 μm), resulting in higher SBS [22,23]. Other studies report comparable SBS values [10] or slightly lower SBS [21,24] with self-etch adhesives, though still within clinically acceptable ranges [10,21,24].

Moisture contamination from saliva or crevicular fluid is a primary cause of bond failure [25]. Hydrophilic adhesives can mitigate this issue, enhancing bonding to moisture-contaminated enamel by incorporating components such as alcohol, which acts as a drying agent, and hydroxyethyl methacrylate (HEMA), which increases wettability, lowers the contact angle, and promotes rapid resin molecules’ spreading [26]. Studies have shown that while hydrophilic adhesives such as OrthoSolo™ (Manufacturer: Ormco Corporation; City: Brea, California; Country: United States) or Assure Plus™ (Manufacturer: Reliance Orthodontic Products, Inc.; City: Itasca; Country: United States) achieve clinically acceptable SBS in wet environments, these values remain significantly lower than in dry conditions [26,27]. This difference is not significant for self-etching adhesives such as Single-Bond [27]. In clinical situations where moisture control is particularly difficult, such as surgical exposure for traction of impacted teeth, acid etching may be undesirable due to potential mucosal irritation. Therefore, further investigation is warranted to assess the effectiveness of self-etch adhesives under saliva contamination, which is a focus of the present study.

Effective adhesion with optimal SBS may leave residual adhesive on the tooth surface after bracket debonding, requiring a cleanup procedure that is time-consuming and may induce iatrogenic effects such as enamel cracks, scratches, or partial loss [6,7,28]. Conversely, if debonding occurs at the adhesive–enamel interface, enamel fractures or cracks may result. Post-debonding enamel evaluation can quantify residual resin and enamel damage using the adhesive remnant index (ARI), where a score of 0 indicates no adhesive remaining on the tooth, 1 indicates less than 50%, 2 indicates more than 50%, and 3 denotes all adhesive remaining with the impression of the bracket base [28].

Given the challenges of maintaining dry conditions during bonding in the molar region, saliva contamination remains a frequent clinical problem in fixed orthodontics. Although numerous in vitro studies have evaluated shear bond strength (SBS) under moisture contamination, the timing of the contamination and the influence of debonding force direction have not been consistently addressed. In particular, the combined effect of saliva contamination and multidirectional loading conditions—which better reflect the clinical environment—remains insufficiently explored. Therefore, an experimental in vitro study with a parallel design was conducted using enamel of extracted human permanent molars and orthodontic tubes to assess the SBS obtained with a conventional adhesive (Transbond XT™—Manufacturer: 3M Unitek (brand of 3M Company), Maplewood, MN, USA), a hydrophilic adhesive (Transbond MIP™—Manufacturer: 3M Unitek (3M Company), Maplewood, MN, USA), and a self-etch adhesive (Scotchbond Universal™ [Manufacturer: 3M Company, Maplewood, MN, USA] used in combination with Transbond XT™ adhesive paste), under dry and saliva-contaminated conditions, as well as to evaluate bond failure patterns using the adhesive remnant index (ARI), enamel integrity, and the effect of debonding force direction (90° or 45° relative to the metallic tube). The primary hypothesis was that adhesive effectiveness, quantified by SBS, would differ according to the presence of saliva contamination, while the secondary hypothesis was that variation in the direction of the applied debonding force would not significantly affect adhesive effectiveness. The main objective of this study was to evaluate whether the shear bond strength (SBS) of orthodontic tubes bonded to human molars in vitro varies according to the adhesive system used, namely a conventional gold-standard adhesive (Transbond XT^TM^), a hydrophilic adhesive (Transbond MIP^TM^), and a self-etch adhesive (Scotchbond Universal^TM^ used in combination with Transbond XT^TM^ adhesive paste), as well as the presence or absence of saliva contamination and the direction of the applied SBS force.

## 2. Materials and Methods

For this in vitro study, patients attending the Dental Hospital of the University of Barcelona who needed extraction of molars were randomly included in the study. After information about the study was given and informed consent was obtained, the extracted teeth were collected by the same investigator (I.M., T) and preserved in deionized water, which was changed at regular intervals to avoid deterioration [29]. The study protocol was approved by the local Ethics Committee (Barcelona University Dental Hospital, nº56/2022). All procedures were carried out following the principles of the Helsinki Declaration. This report follows a modified approach to the Consolidated Standards of Reporting Trials (CONSORT) guidelines adapted for in vitro studies on dental materials (Supplementary File S1) [30].

**Figure 1:**
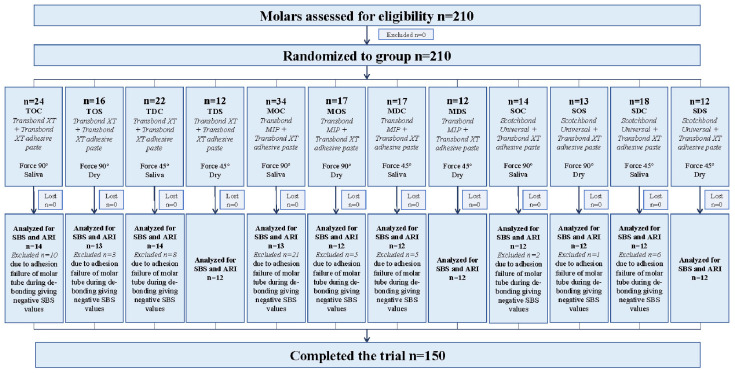
CONSORT diagram.

Molars without any caries, restorations and cracks on the buccal enamel surface were collected. The minimum sample size was determined (120 molars) based on the recommendations of Streiner et al., where a ratio of 10:1 is suggested for each variable studied [31]. The teeth were cleaned with ultrasound and a prophylaxis brush, thoroughly washed with water and air-dried. The samples were randomly divided into 12 groups by the same investigator (I.M., T) as summarized in Figure 1—CONSORT diagram. No blinding methods were applied.

**Bonding procedure**

Roth molar tubes (Forestadent Confort Line Roth-System, slot 0.022” × 0.028”, Bernhard Förster GmbH, Pforzheim, Germany), with a base surface area of 14.98 mm^2^, were bonded to tooth samples as follows (Table 1):

1. Group 1 (TOS) and group 2 (TDS): the enamel surface was etched with 37% phosphoric acid gel (3M Healthcare Unitek, Monrovia, CA, USA) for 20 s, washed with water spray for 30 s, and air-dried with a moisture-free air blower to a chalky white appearance. Transbond XT^TM^ (3M Healthcare Unitek, Monrovia, CA, USA) was then applied to the etched surface and dried with air for 5 s. Transbond XT^TM^ adhesive paste (3M Healthcare Unitek, Monrovia, CA, USA) was placed on molar tubes (Forestadent Confort Line Roth-System, slot 0.022” × 0.028”, Bernhard Förster GmbH, Pforzheim, Germany); the tube was bonded to the tooth surface perpendicular to the long axis of the buccal surface. In all groups, the tubes were light-cured by an LED light-curing unit (Woodpecker medical instrument Co., Ltd. Information Industrial Park, National High-Tech Zone, Guangxi, China) at a light intensity of 1000 mW/cm^2^ for a total of 20 s with the light beam directed for 10 s at each of the mesial and distal faces. All the procedures were carried out according to the manufacturers’ instructions.
2. Group 3 (TOC) and group 4 (TDC): the procedure was performed in the same way as group 1 and 2; however, before and after the application of the adhesive, the surface of the tooth was contaminated with a thin layer of artificial saliva (Marti Mas, Barcelona, Spain), left on the surface for 10 s, and then gently air dried for 5 s with a moisture-free air blower. The artificial saliva had the following composition (per 100 mL): carmellose sodium 1.00 g, sorbitol 3.00 g, potassium chloride 0.12 g, potassium dihydrogen phosphate 0.034 g, sodium chloride 0.084 g, anhydrous calcium chloride 0.015 g, and magnesium chloride hexahydrate 0.005 g; purified water was used as excipient to make up to 100 mL (c.s.p.).
3. Group 5 (MOS) and group 6 (MDS): the procedure was performed in the same way as group 1 and 2, but using Transbond MIP^TM^ (3M Healthcare Unitek, Monrovia, CA, USA).
4. Group 7 (MOC) and group 8 (MDC): the procedure was performed in the same way as group 3 and 4, but using Transbond MIP^TM^ (3M Healthcare Unitek, Monrovia, CA, USA).
5. Group 9 (SOS) and group 10 (SDS): Scotchbond Universal^TM^ (3M Healthcare ESPE, Seefeld, Germany) was rubbed onto the enamel surface for 20 s according to the manufacturer’s instructions, and the enamel surface was gently dried by a moisture-free air blower. The surface was light-cured for 10 s before bonding the tube, in the same way as in group 1 and 2.
6. Group 11 (SOC) and group 12 (SDC): the procedure was performed in the same way as group 9 and 10, however before and after the application of the primer, the surface of the enamel was contaminated with artificial saliva (Marti Mas, Barcelona, Spain), left on the surface for 10 s, and then gently air dried for 5 s with a moisture-free air blower.

**Inclusion in acrylic resin and debonding procedure**

**Figure 2:**
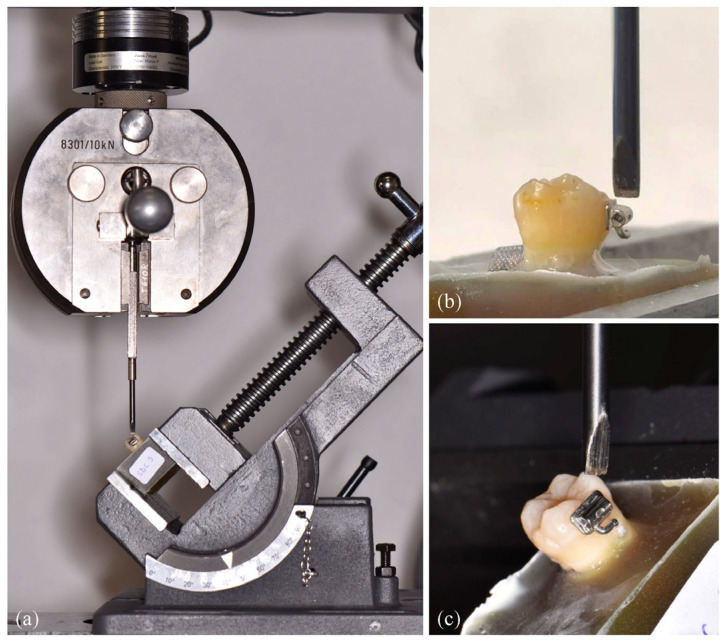
Debonding procedure. (**a**) electromechanical universal testing machine (ZwickRoell, Ulm, Germany); (**b**) 90° force application; (**c**) 45° force application.

Bonded samples were positioned in acrylic resin blocks, up to 1 mm apical to the cement-enamel junction, in such a way that the tubes were perpendicular to the shear blade. The debonding procedure was performed at the Faculty of Chemistry, University of Barcelona, Spain. SBS was evaluated with the specimens placed in an inclinable vise attached to the base plate of an electromechanical universal testing machine (ZwickRoell, Ulm, Germany), and the force was applied to the tube-tooth interface with a blade (Figure 2). In groups 1, 3, 5, 7, 9 and 11 the force was applied at an angle of 90°, and in groups 2, 4, 6, 8, 10 and 12 at 45°, at a crosshead speed of 10 mm/minute until the tube was detached. The maximum force at bond failure of the tubes from the tooth surface was recorded in newtons (N), and the SBS was determined in mega pascals (MPa) by dividing the force by the tube surface area (14.98 mm^2^). Shear bond strength (MPa) = stress at failure (N)/cross-sectional surface area of the tube base (mm^2^).

**Assessment of adhesive remnant index (ARI)**

**Figure 3:**
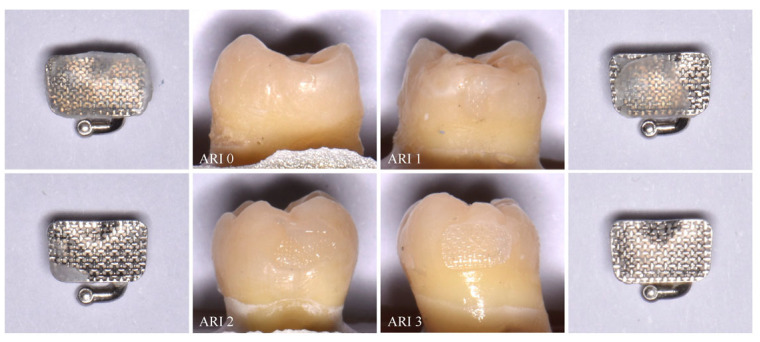
Adhesive Remnant Index (ARI); molars with their corresponding tubes.

Once the tubes had been debonded, the enamel surface of each tooth and each tube base were analyzed using an optical microscope under 10× magnification and were also photographed using a digital camera (Olympus OM-D E-M5 Mark III, Olympus Corp., Tokyo, Japan), a macro lens (Olympus M.Zuiko ED 60 mm f/2.8 Macro, Olympus Corp., Tokyo, Japan), and a twin flash (Olympus STF-8 Macro Twin Flash, Olympus Corp., Tokyo, Japan), to determine the adhesive remnant on the surface (Figure 3). The ARI scale was used to classify the enamel surface after debonding [28]:

0: no adhesive left on the tooth.

1: less than 50% of the adhesive left on the tooth.

2: more than 50% of the adhesive left on the tooth.

3: all adhesive left on the tooth, with an impression of the bracket base mesh.

**Data Analysis**

The descriptive statistics were calculated for SBS and ARI values. The Kruskal–Wallis test for adhesive and ARI and the Mann–Whitney test for force direction and saliva were applied to determine any significant differences in SBS values among groups. The distribution of failure modes was analyzed using the Chi-square test. The level of significance was predetermined at the 95% confidence level (*p* < 0.05). All the statistical analyses were conducted using the SPSS 23.0 package for Windows (SPSS Inc., Chicago, IL, USA).

## 3. Results

The normality of the data was assessed using the Shapiro–Wilk test, concluding that SBS does not follow a normal distribution (*p*-value < 0.001). The histogram analysis revealed a bimodal distribution, with two peaks corresponding to measurements taken with and without saliva. Therefore, the results were stratified based on this variable. Consequently, non-parametric tests were employed for mean comparisons.

The Kruskal–Wallis and Mann–Whitney tests indicated a greater variability in SBS values in samples with saliva contamination compared to dry conditions, concerning both the type of adhesive and the direction of the applied force.

When analyzing the adhesives divided according to the presence or absence of saliva, the Kruskal–Wallis test demonstrated no statistically significant differences (*p* > 0.05) in the SBS generated during the debonding of tubes between the three evaluated adhesives. However, all adhesives in dry conditions presented a significantly higher SBS (*p* < 0.001) than with saliva, and the same results were obtained when each adhesive was compared separately (Table 2).

When results were stratified according to saliva contamination, and the effect of force applied at 90° or 45° for each adhesive was analyzed, no statistically significant differences (*p* > 0.05) were observed among the adhesives used under saliva contamination. In dry conditions, overall, SBS was significantly higher (*p* < 0.05) when forces were applied at 45°. Moreover, Scotchbond Universal^TM^ adhesive required a statistically greater SBS when force was applied at 45° (Table 3).

When we analyze ARI, we see that when all the adhesives are observed in total, a higher frequency of specimens has an ARI 3 in dry conditions, and an ARI 0 in the group with saliva present. Evaluating adhesives separately, we see that in dry conditions, only Scotchbond Universal^TM^ is different, with a higher percentage of ARI 1, while with saliva present, only Transbond XT^TM^ differs, with the largest frequency of an ARI 1 (Table 3). Comparing ARI scores among the groups, the Chi-square test did not reveal significant differences between the groups (Table 4).

When analyzing the relationship between the ARI score and the SBS, in the group with saliva contamination, the Kruskal–Wallis test revealed a significant difference in the SBS (*p* = 0.05) and no significant differences in the non-contaminated group (Table 5). The Kruskal–Wallis test showed no significant differences in ARI scores when analyzing the results according to saliva contamination and applied force direction (*p* > 0.05) (Table 6).

## 4. Discussion

In orthodontic practice, enhancing efficiency often involves reducing chair time by simplifying the bonding procedure without compromising bond strength. We seek minimally invasive techniques that ensure the best possible adhesion even under challenging conditions, such as moisture contamination and high masticatory and orthodontic forces. Achieving a dry environment for bonding can be difficult, especially in posterior regions or during surgical exposure of impacted teeth. Various strategies have been proposed to address these challenges, including the use of hydrophilic adhesives that maintain bond strength in the presence of moisture [2,25] and self-etch adhesives, which simplify enamel conditioning by eliminating the acid-etching step. This reduction in procedural steps not only decreases the risk of moisture contamination but also minimizes aerosol generation and potential damage to both enamel and surrounding tissues associated with 37% phosphoric acid etching [15].

Our multicentric study suggests that in dry conditions, the SBS of orthodontic tubes bonded to human molars in vitro does not differ significantly between conventional gold-standard orthodontic adhesives like Transbond XT^TM^, hydrophilic adhesives like Transbond MIP^TM^, and self-etch adhesives like Scotchbond Universal^TM^ when used in combination with Transbond XT^TM^ adhesive paste. For both Transbond XT™ and Transbond MIP™, the enamel surface was pre-etched, whereas Scotchbond Universal™ was applied directly to the enamel. These findings suggest that eliminating the acid-etch step does not adversely affect the bond strength of Scotchbond Universal^TM^ in dry conditions. This observation is consistent with the results of Arash V. et al. and Griffin J. et al. [10,24], but contrasts with Sharma et al., who reported that self-etch adhesives achieve lower, albeit clinically acceptable, SBS values [21]. Consequently, Scotchbond Universal™, when used as a self-etch adhesive, may be advantageous in clinical scenarios where acid etching poses a risk—such as near surgical incisions—or where saliva control is suboptimal and procedural simplification is desired. Further research is warranted to assess its performance in the presence of blood contamination and to determine whether prior acid etching could further enhance its bond strength.

Saliva contamination is a well-recognized factor contributing to bond failure [25], reducing SBS values for all tested adhesives compared to dry conditions, consistent with previous studies [26,27]. Under saliva contamination, all three adhesives exhibited similar behavior; however, while SBS values in dry conditions approach clinically acceptable thresholds [6], they fall substantially below acceptable levels in the presence of moisture and display greater variability. This suggests that saliva affects the adhesion capacity unevenly, resulting in clinically unpredictable bracket bonding.

Scanning electron microscopy studies have demonstrated that saliva can occlude the porosities created by acid etching, thereby reducing micromechanical retention through resin tag formation and consequently decreasing SBS [21,27,32]. In conventional etch-and-rinse systems, phosphoric acid removes the smear layer and opens enamel tubules, making the substrate particularly susceptible to contamination, as saliva can penetrate the etched surface and interfere with resin infiltration. Enamel surfaces may therefore become contaminated not only before bonding but also during etching or after adhesive application [33]. In contrast, in self-etch systems such as Scotchbond Universal™, adhesives are applied without prior acid etching, saliva contamination occurring only before adhesive application—as performed in previous studies—may have a less pronounced effect, as the enamel tubules remain largely closed, and would not adequately simulate clinical conditions [26,27]. Therefore, in this study, saliva contamination was performed both before and after adhesive application for all adhesives, to ensure a comparable and clinically relevant contamination challenge and to better simulate the clinical situation, where contamination may occur at multiple stages of the bonding procedure, particularly during posterior bonding [26,27].

Another aspect of bracket debonding that has not received attention in previous research is the influence of debonding force direction. The complexity of the oral environment cannot be fully reproduced using an electromechanical universal testing machine [32], as bonded orthodontic attachments—particularly molar tubes—are exposed to multidirectional forces arising from mastication, occlusal interferences, and orthodontic mechanics. In addition, posterior teeth frequently present buccolingual and mesiodistal inclinations, which may result in oblique force vectors acting on bonded appliances rather than purely perpendicular loading. In this study, we evaluated the effect of forces applied both perpendicular (90°) and inclined (45°) to the tube. In dry conditions, SBS was higher at 45° than at 90°. Under saliva contamination, only Scotchbond Universal™ exhibited a statistically significant increase in SBS at 45° (*p* < 0.05). These findings suggest that greater dental crowding, resulting in tooth inclinations up to 45°, may not reduce resistance to debonding forces, as indicated by the greater SBS values. From a clinical perspective, this may help explain why appliance failure in molar regions cannot be attributed solely to moisture contamination or adhesive selection but may also be influenced by the direction and distribution of occlusal loading. Nonetheless, confirmation of these results may require larger sample sizes or split-mouth in vivo studies.

In restorative dentistry, higher bond strength is generally desirable; however, in orthodontics, an optimal bond strength is required, sufficient to maintain bracket and enamel integrity during treatment but low enough to allow safe debonding without enamel damage [34]. Reynolds et al. established an ideal range of 5.9 to 7.8 MPa for clinically successful bonding [6]. Nevertheless, these values warrant careful interpretation, as Reynolds’ findings were obtained from in vivo studies, whereas the present research was conducted in vitro. Accordingly, the fact that many SBS values obtained under saliva contamination in the present study fell below the traditionally cited clinical threshold should not be interpreted as indicating clinically unacceptable adhesive performance. Rather, these findings reflect relative differences in bonding behavior under standardized conditions. Direct extrapolation of in vitro SBS values to clinical success or failure should therefore be avoided. Comparisons with previous studies should therefore be made cautiously, since SBS can be influenced by multiple factors, including the bracket or tube material, base mesh design, adhesive type, sample storage conditions, thermocycling, testing apparatus, debonding force direction, crosshead speed, blade geometry, and variations in bonding and debonding procedures [24,27,35]. Previous studies for conventional (Transbond XT^TM^) [7,11,21,24,36], hydrophilic (OrthoSolo^TM^, Assure Plus^TM^) [26,27] or self-etch adhesives (Transbond XT Plus^TM^, G-Premio Bond^TM^, Xeno V^TM^) (G-Premio Bond^TM^: Manufacturer: GC Europe N.V., City: Leuven, Country: Belgium; Xeno V^TM^: Manufacturer: Dentsply, City: Konstanz, Country: Germany) show comparable but generally higher SBS values than those obtained in the present study [10,21,24]. Although many SBS values obtained under saliva contamination were below the traditionally cited clinical threshold of 5.9–7.8 MPa, these findings should not be interpreted as indicating clinically unacceptable performance. The Reynolds threshold was derived from in vivo observations, whereas in vitro SBS testing serves primarily as a comparative tool under controlled conditions. Therefore, SBS values should be interpreted comparatively within the same experimental framework rather than directly extrapolated to clinical success or failure.

To complement SBS analysis, the adhesive remnant index (ARI) provides a visual assessment of the quality of adhesion and the site of bond failure—whether between the adhesive and enamel or between the adhesive and bracket base [26,28]. An ARI score of 0 indicates adhesive failure, at the enamel–adhesive interface, whereas a score of 3 denotes cohesive failure, within the adhesive–bracket interface [6,16,26]. Although higher ARI scores are associated with increased chair time for the clinician, they are preferable as they minimize the risk of enamel damage during debonding [6,17,26].

In our study, adhesives applied under dry conditions exhibited a higher frequency of ARI score 3, whereas the presence of saliva resulted in more frequent ARI 0 scores. Specifically, Transbond XT™ and Transbond MIP™ showed predominantly ARI 3, while Scotchbond Universal™ tended toward ARI 1. These findings are consistent with previous reports for Transbond XT^TM^ [7,26], hydrophilic [10,26], and self-etch adhesives [10,21,24]. The frequent occurrence of ARI 3 for both conventional and hydrophilic adhesives, and the smaller amount of adhesive remaining on enamel with self-etch adhesives, may be attributed to the use of 37% phosphoric acid etching. Enamel surfaces appear more porous following acid etching, whereas those treated with self-etch adhesives present smoother and cleaner surfaces after debonding [21]. Under saliva contamination, Transbond XT™ primarily showed ARI 1, and both Transbond MIP™ and Scotchbond Universal™ exhibited ARI 0, in agreement with previous studies on conventional [26], hydrophilic [26,27], and self-etch adhesives [10,21,24]. This pattern reflects the interference of saliva with mechanical adherence by obstructing enamel microporosities and reducing resin tag formation [21,27,32].

Some studies report an ARI 1–2 for Transbond XT^TM^ [7,11,21,24] and hydrophilic adhesives [7,27] in dry conditions. ARI scoring, determined visually, depends on several factors—including examiner subjectivity, bracket base design, and adhesive type—but appears to be largely independent of SBS values [27,37]. In our study, under saliva contamination, SBS tended to increase from ARI 0 to ARI 2 (corresponding to the highest SBS values) and then decrease at ARI 3 (lowest SBS values), indicating that mixed cohesive–adhesive failures were associated with higher bond strength. This finding, although it may appear counterintuitive, may be explained by the fact that the enamel–resin bond generally requires a higher force to fail than the resin–metal interface, resulting in residual adhesive on the enamel surface even in the presence of saliva contamination [38]. When analyzed by debonding force direction, these differences were not statistically significant. This outcome may be attributed to the greater variability of SBS values in the presence of saliva. Because adhesives are designed to infiltrate enamel, while saliva is not, the uneven occlusion of enamel porosities by saliva likely leads to irregular contamination patterns and inconsistent resin tag formation.

This study has certain limitations that should be acknowledged. Clinical extrapolation of in vitro findings should be made cautiously, as such studies provide only preliminary guidance for material selection [39]. The oral environment can significantly influence material biodegradation, altering their composition, mechanical behavior, and, consequently, their bond strength—factors that cannot be fully replicated in vitro [40,41]. To better simulate clinical conditions, future research should assess debonding within two hours of bonding to approximate the timing of initial wire placement [7]. Additionally, increasing sample size could help achieve a normal distribution and permit the use of parametric statistical analyses.

## 5. Conclusions

All three adhesives—conventional, hydrophilic, and self-etch—demonstrated similar shear bond strength (SBS) when tested under identical conditions, but significantly higher SBS in dry environments than in saliva-contaminated ones. This supports the use of self-etch adhesives in dry conditions without compromising bond strength.

Under saliva contamination, the direction of debonding force (45° vs. 90°) did not significantly affect SBS. However, in dry conditions, applying force at 45° increased SBS across all adhesives, particularly with Scotchbond Universal™.

A higher frequency of ARI score 3 (adhesive remaining on enamel) was observed under dry conditions, while ARI 0 (adhesive remaining on bracket) predominated in saliva-contaminated groups. Although this distribution was not statistically significant, it may reflect reduced resin tag formation in the presence of moisture.

Notably, under saliva contamination, specimens with an ARI score of 0 exhibited higher SBS than those with ARI 3, while ARI scores 1 and 2 were associated with the highest bond strengths.

These findings emphasize the importance of moisture control and bonding angle and suggest that ARI scores may reflect complex failure modes under contamination. Such variables should be considered when selecting adhesives and protocols for molar bonding in clinical orthodontics.

## Figures and Tables

**Table 1:** Groups tested.

Group	Code	Material Used	Contamination	Force Direction
1	TOC (n = 14)	Transbond XT primer + Transbond XT adhesive	Saliva contamination	90°
2	TOS (n = 13)	Transbond XT primer + Transbond XT adhesive	Dry conditions	90°
3	TDC (n = 14)	Transbond XT primer + Transbond XT adhesive	Saliva contamination	45°
4	TDS (n = 12)	Transbond XT primer + Transbond XT adhesive	Dry conditions	45°
5	MOC (n = 13)	Transbond MIP primer + Transbond XT adhesive	Saliva contamination	90°
6	MOS (n = 12)	Transbond MIP primer + Transbond XT adhesive	Dry conditions	90°
7	MDC (n = 12)	Transbond MIP primer + Transbond XT adhesive	Saliva contamination	45°
8	MDS (n = 12)	Transbond MIP primer + Transbond XT adhesive	Dry conditions	45°
9	SOC (n = 12)	Scotch Bond Universal + Transbond XT adhesive	Saliva contamination	90°
10	SOS (n = 12)	Scotch Bond Universal + Transbond XT adhesive	Dry conditions	90°
11	SDC (n = 12)	Scotch Bond Universal + Transbond XT adhesive	Saliva contamination	45°
12	SDS (n = 12)	Scotch Bond Universal + Transbond XT adhesive	Dry conditions	45°

**Table 2:** Descriptive statistics and Kruskal–Wallis and Mann–Whitney analysis for all experimental groups. SBS, shear bond strength; SD, standard deviation; T, Transbond XT; M, Transbond MIP; S, Scotchbond Universal.

		SBS (MPa)					
	Adhesive	Mean	SD	Median	(P25–P75)	(Min–Max)	*p*-Value ^1^
Dry conditions	Total of adhesives (n = 73)	5.6	(1.3)	5.7	(5.3; 6.0)	(0.8; 8.5)	
	T (n = 25)	5.4	(0.9)	5.7	(5.2; 6.0)	(3.5; 6.6)	0.927
	M (n = 24)	5.7	(1.6)	5.7	(5.2; 6.8)	(2.2; 8.5)	
	S (n = 24)	5.5	(1.4)	5.7	(5.4; 6.0)	(0.8; 7.9)	
Saliva contamination	Total of adhesives (n = 77)	4.1	(1.4)	4.2	(3.3; 5.3)	(0.7; 6.5)	
	T (n = 28)	4.1	(1.5)	4.1	(3.5; 5.4)	(0.9; 6.3)	0.130
	M (n = 25)	3.6	(1.5)	4.0	(2.2; 4.5)	(0.7; 6.5)	
	S (n = 24)	4.5	(1.1)	4.6	(3.9; 5.3)	(2.0; 6.1)	

^1^ Kruskal–Wallis test for adhesive and Mann–Whitney analysis for saliva.

**Table 3:** Relationship between applied force direction and SBS for all experimental groups.

			SBS (MPa)					
	Adhesive	Force Direction	Mean	SD	Median	(P25–P75)	(Min–Max)	*p*-Value ^1^
Dry conditions	Total of adhesives	O (n = 37)	5.1	(1.3)	5.5	(4.5; 5.9)	(0.8; 7.1)	0.025 *
		D (n = 36)	6.0	(1.2)	5.8	(5.5; 6.7)	(3.7; 8.5)	
	T	O (n = 13)	5.3	(1.0)	5.9	(4.5; 5.9)	(0.8; 7.1)	0.810
		D (n = 12)	5.6	(0.7)	5.6	(4.5; 5.9)	(0.8; 7.1)	
	M	O (n = 12)	5.0	(1.4)	5.6	(4.1; 5.8)	(2.2; 7.1)	0.089
		D (n = 12)	6.3	(1.5)	6.6	(5.4; 7.5)	(3.9; 8.5)	
	S	O (n = 12)	5.0	(1.5)	5.4	(4.8; 5.9)	(0.8; 6.0)	0.028 *
		D (n = 12)	6.1	(1.0)	5.8	(5.7; 6.7)	(4.1; 7.9)	
Saliva contamination	Total of adhesives	O (n = 39)	4.0	(1.6)	4.1	(2.7; 5.3)	(0.7; 6.5)	0.768
		D (n = 38)	4.1	(1.3)	4.3	(3.6; 5.0)	(0.6; 6.3)	
	T	O (n = 14)	4.1	(1.4)	4.2	(3.3; 5.3)	(1.1; 6.2)	0.946
		D (n = 14)	4.1	(1.6)	4.1	(3.6; 5.5)	(0.9; 6.3)	
	M	O (n = 13)	3.4	(2.0)	3.3	(1.9; 5.4)	(0.7; 6.5)	0.574
		D (n = 12)	3.9	(0.8)	4.1	(3.2; 4.4)	(2.1; 5.0)	
	S	O (n = 12)	4.5	(1.0)	4.4	(3.8; 5.3)	(2.7; 6.1)	0.887
		D (n = 12)	4.5	(1.3)	4.6	(3.9; 5.5)	(2.0; 5.8)	

^1^* p*-value for O vs. D (Mann–Whitney test). * *p*-value ≤ 0.05.

**Table 4:** Adhesive remnant index (ARI) scores for all experimental groups. SBS, shear bond strength; SD, standard deviation; T, Transbond XT; M, Transbond MIP; S, Scotchbond Universal.

	Adhesive	ARI 0	ARI 1	ARI 2	ARI 3
Dry conditions	Total of adhesives (n = 73)	15% (n = 11)	27% (n = 20)	21% (n = 15)	37% (n = 27)
	T (n = 25)	16% (n = 4)	34% (n = 6)	24% (n = 6)	36% (n = 9)
	M (n = 24)	4% (n = 1)	17% (n = 4)	25% (n = 6)	54% (n = 13)
	S (n = 24)	25% (n = 6)	42% (n = 10)	13% (n = 3)	21% (n = 5)
Saliva contamination	Total of adhesives (n = 77)	36% (n = 28)	30% (n = 23)	12% (n = 9)	22% (n = 17)
	T (n = 28)	25% (n = 7)	36% (n = 10)	11% (n = 3)	29% (n = 8)
	M (n = 25)	32% (n = 8)	24% (n = 6)	20% (n = 5)	24% (n = 6)
	S (n = 24)	54% (n = 13)	29% (n = 7)	4% (n = 1)	13% (n = 3)

**Table 5:** Relationship between ARI and SBS.

			SBS (MPa)					
		AIR	Mean	SD	Median	(P25–P75)	(Min–Max)	*p*-Value ^1^
Dry conditions	0	(n = 11)	5.8	1.3	5.7	(5.5; 6.6)	(3.8; 7.9)	0.154
	1	(n = 20)	6.0	0.7	5.9	(5.5; 6.0)	(5.2; 8.1)	
	2	(n = 15)	5.9	0.9	5.9	(5.3; 6.1)	(3.9; 7.7)	
	3	(n = 27)	5.0	1.6	5.5	(4.1; 5.9)	(0.8; 8.5)	
Saliva contamination	0	(n = 28)	3.9	1.3	4.1	(3.2; 4.7)	(0.9; 5.8)	0.058 *
	1	(n = 23)	4.2	1.6	4.4	(3.6; 5.5)	(0.9; 6.3)	
	2	(n = 9)	5.0	1.4	5.7	(4.0; 5.8)	(2.2; 6.5)	
	3	(n = 17)	3.5	1.4	3.5	(2.9; 4.3)	(0.7; 6.2)	

^1^ Kruskal–Wallis test for SBS and ARI. * *p*-value ≤ 0.05

**Table 6:** Relationship between ARI, force direction and SBS.

				SBS (MPa)					
	Force Direction		AIR	Mean	SD	Median	(P25–P75)	(Min–Max)	*p*-Value ^1^
Dry conditions	O	0	(n = 3)	4.4	1.0	4.0	(3.8; 5.5)	(3.8; 5.5)	0.077
		1	(n = 7)	5.6	0.3	5.5	(5.4; 5.9)	(5.2; 6.0)	
		2	(n = 7)	6.1	0.5	6.0	(5.9; 6.0)	(5.7; 7.1)	
		3	(n = 20)	4.7	1.5	5.4	(3.9; 5.8)	(0.8; 6.2)	
	D	0	(n = 8)	6.3	1.0	5.7	(5.6; 7.1)	(5.5; 7.9)	0.491
		1	(n = 13)	6.2	0.8	5.9	(5.7; 6.4)	(5.4; 8.1)	
		2	(n = 8)	5.7	1.2	5.5	(5.1; 6.5)	(3.9; 7.7)	
		3	(n = 7)	5.6	1.8	5.7	(4.1; 6.7)	(3.7; 8.5)	
Saliva contamination	O	0	(n = 10)	3.7	1.6	3.9	(2.7; 4.8)	(0.9; 5.7)	0.314
		1	(n = 8)	4.4	1.8	5.3	(3.0; 6.7)	(1.1; 6.1)	
		2	(n = 7)	4.7	1.4	4.7	(4.0; 5.8)	(2.2; 6.5)	
		3	(n = 14)	3.6	1.5	3.6	(3.2; 4.5)	(0.7; 6.2)	
	D	0	(n = 18)	4.1	1.1	4.2	(3.7; 4.7)	(1.6; 5.8)	0.094
		1	(n = 15)	4.2	1.5	4.4	(3.6; 5.5)	(0.9; 6.3)	
		2	(n = 2)	5.9	0.4	5.9	(5.7; 6.2)	(5.7; 6.2)	
		3	(n = 3)	3.1	0.9	2.9	(2.3; 4.1)	(2.3; 4.1)	

^1^ Kruskal–Wallis test.

## Data Availability

The original contributions presented in the study are included in the article, further inquiries can be directed to the corresponding author.

## Supplementary Materials

The following supporting information can be downloaded at: https://www.mdpi.com/article/10.3390/jfb17020089/s1, File S1: CONSORT guidelines checklist.

## Abbreviations

The following abbreviations are used in this manuscript:

General Abbreviations:	
ARI	Adhesive remnant index
CONSORT	Consolidated Standards of Reporting Trials
HEMA	Hydroxyethyl methacrylate
LED	Light-emitting diode
MPa	Megapascal
N	Newton
SBS	Shear bond strenght
SPSS	Statistical Package for the Social Sciences
Adhesive Codes:	
T	Transbond XT (conventional adhesive)
M	Transbond MIP (hydrophilic adhesive)
S	Scotchbond Universal (self-etch adhesive)
Condition and Force Direction Codes:	
O	Dry (no saliva contamination)
C	Saliva-contaminated
S	Force applied at 90°
D	Force applied at 45°
