# Supplementary material for: Efficiency of Orthodontic Adhesives: Influence of Saliva and Shear Direction—In Vitro Study
Source: J Funct Biomater. 2026 Feb 11;17(2):89. doi: 10.3390/jfb17020089 (PMC12941484; doi:10.3390/jfb17020089)
Supplement: Supplementary file 1 [file jfb-17-00089-s001.zip › jfb-4083224-supplementary.pdf]

Supplementary File: *CONSORT Checklist*

Section/topic	Checklist item	Page
**Abstract**	• Item 1. Structured summary of trial design, methods, results, and conclusions	Pg. 3<br>Pg. 5
**Introduction**		
Background and objectives	• Item 2a. Scientific background and explanation of rationale<br>• Item 2b. Specific objectives and/or hypotheses	Pg. 9-11<br>Pg. 11
**Methods**		
Intervention	• Item 3. The intervention for each group, including how and when it was administered, with sufficient detail to enable replication	Pg. 11-16
Outcomes	• Item 4. Completely defined, pre-specified primary and secondary measures of outcome, including how and when they were assessed	Pg. 11-15
Sample size	• Item 5. How sample size was determined	Pg. 12
Randomization:		
Sequence generation	• Item 6. Method used to generate the random allocation sequence	Pg. 12
Allocation concealment mechanism	• Item 7. Mechanism used to implement the random allocation sequence (for example, sequentially numbered containers), describing any steps taken to conceal the sequence until intervention was assigned	Not applicable<br>Pg. 12
Implementation	• Item 8. Who generated the random allocation sequence, who enrolled teeth, and who assigned teeth to intervention	Pg. 12
Blinding	• Item 9. If done, who was blinded after assignment to intervention (for example, care providers, those assessing outcomes), and how	Pg. 12
Statistical methods	• Item 10. Statistical methods used to compare groups for primary and secondary outcomes	Pg. 16
**Results**		
Outcomes and estimation	• Item 11. For each primary and secondary outcome, results for each group, and the estimated size of the effect and its precision (for example 95% confidence interval)	Pg. 16-19
**Discussion**		
Limitations	• Item 12. Trial limitations, addressing sources of potential bias, imprecision, and, if relevant, multiplicity of analyses	Pg. 20<br>Pg. 22
**Other information**		
Funding	• Item 13. Sources of funding and other support (for example suppliers of drugs), role of funders	Pg. 23
Protocol	• Item 14. Where the full trial protocol can be accessed, if available	Pg. 12
